# Sex differences in systemic sclerosis: from pathogenesis to clinical manifestations and treatment

**DOI:** 10.1177/1759720X251384602

**Published:** 2025-10-14

**Authors:** Melpomeni Toitou, Maria Iacovantuono, Gesa Sauer, Carina Mihai, Maria Sole Chimenti, Oliver Distler, Cosimo Bruni, Muriel Elhai

**Affiliations:** Department of Rheumatology, University Hospital of Zurich, University of Zurich, Rämistrasse 100, Zurich 8091, Switzerland; Department of Rheumatology, University Hospital of Zurich, University of Zurich, Zurich, Switzerland; Rheumatology, Allergology and Clinical Immunology, Department of Systems Medicine, Tor Vergata University, Rome, Italy; Department of Rheumatology, University Hospital of Zurich, University of Zurich, Zurich, Switzerland; Department of Rheumatology, University Hospital of Zurich, University of Zurich, Zurich, Switzerland; Rheumatology, Allergology and Clinical Immunology, Department of Systems Medicine, Tor Vergata University, Rome, Italy; Department of Rheumatology, University Hospital of Zurich, University of Zurich, Zurich, Switzerland; Department of Rheumatology, University Hospital of Zurich, University of Zurich, Zurich, Switzerland; Department of Rheumatology, University Hospital of Zurich, University of Zurich, Zurich, Switzerland

**Keywords:** epidemiology, gender, pathogenesis, prognosis, sex, sex differences, systemic sclerosis, treatment response

## Abstract

Systemic sclerosis (SSc) exhibits sex-related disparities in prevalence, clinical features, and outcomes. While women are more frequently affected, men often experience a more severe disease course, including diffuse cutaneous involvement, interstitial lung disease, and reduced survival. These differences are shaped by biological factors such as sex hormones and genetic influences. Estrogens and androgens differentially influence immune and fibrotic pathways, while life stages such as menopause further modulate disease expression. Genetic mechanisms, including X chromosome inactivation, regulation of immune-related genes, and cell signaling pathways, vary between sexes and also play an important role in the sex bias seen in SSc. In addition to these biological aspects, gender, as a sociocultural factor involving roles, behaviors, and access to care, may further modify disease perception, healthcare engagement, and outcomes, though it remains underexplored in SSc research. Treatment responses may also vary by sex, as suggested by emerging studies, but sex-specific clinical recommendations are still lacking. This review aims to summarize current knowledge on sex-related differences in SSc and highlight implications for clinical management and future research.

## Plain language summary

Understanding how sex influences Systemic Sclerosis: From underlying causes to symptoms and treatment

Systemic sclerosis (SSc) is a complex autoimmune disease that affects the skin and internal organs. It is more common in women than in men, but men often experience a more severe disease course. This includes greater organ involvement and poorer overall outcomes. Biological factors, such as sex hormones and genetic differences, are thought to play a central role in these disparities. Estrogens and androgens affect immune responses and the development of fibrosis in different ways. Changes in hormone levels across life stages, such as during menopause, can also influence how the disease appears. Genetic mechanisms, including how genes on the X chromosome are expressed, contribute further to the differences seen between men and women. In addition to these biological influences, gender-related factors, such as differences in social roles, health behaviors, and access to care, may also affect how people experience the disease and respond to treatment. However, these aspects are still poorly studied in SSc. This review outlines the current understanding of how sex shapes the development, clinical presentation, and treatment responses in SSc, and emphasizes the need for more personalized approaches in both research and clinical care.

## Introduction

Understanding the complex nature of autoimmune diseases requires attention to sex as a fundamental biological variable potentially contributing to disease development, progression, and outcomes.^1^ Among autoimmune diseases, systemic sclerosis (SSc) has a strong female predominance and is characterized by vascular changes, immune dysregulation and fibrosis involving the skin and internal organs.^2–5^ SSc is generally classified into two types: limited cutaneous (lcSSc) and diffuse cutaneous (dcSSc), the latter characterized by more widespread skin fibrosis. Most patients present with antinuclear antibodies (ANAs), but several specific autoantibodies can help identify the disease, such as anticentromere antibodies (ACAs), antitopoisomerase I (anti-Scl70), and anti-RNA polymerase III antibodies.

On top of the strong female predominance of SSc, biological sex differences are known to influence immune responses, vascular health, and fibrosis—all key elements in SSc pathogenesis.^6^ In parallel, sex-related factors, including health behaviors, occupational exposures, and access to care, may further modify disease expression.^6,7^

Despite advances in treatment, SSc remains associated with substantial morbidity and mortality.^8^ Therefore, a better understanding of how sex affects SSc is essential for unraveling its complex pathogenesis, improving risk stratification, and developing sex-adapted therapeutic strategies. This review aims to provide an overview of the influence of sex on SSc, with a focus on epidemiology, underlying pathophysiology, clinical phenotype, disease progression and treatment response.

## Sex differences in the epidemiology of SSc

SSc, like other autoimmune diseases, predominantly affects women. Two large meta-analyses reported pooled prevalence values of 17.6 and 18.87 per 100,000 individuals, respectively. Both studies found a substantially higher prevalence in women—28.0 and 31.2 per 100,000—compared to 6.0 and 6.8 per 100,000 in men, respectively.^2,3^ This translates into an average female-tomale ratio around 5:1.^2^

The prevalence of SSc varies between countries, reflecting both geographic and methodological differences, consistent though, sex disparities. Reported female-to-male ratios range from 4:1 to 14.5:1, with the highest observed in Japan (14.5:1).^2,9–15^ Generally, the prevalence ratio peaks during the reproductive years^6^; however, a Latvian cohort reported a higher gender ratio among individuals aged 70–79 years.^13^

Incidence rates also show substantial variation across regions. Bairkdar et al. estimated a pooled global incidence of 1.4 per 100,000 person-years, with women affected more often than men (2.3 vs 0.5 per 100,000).^2^ A more recent meta-analysis reported a higher global incidence of 8.64 per 100,000 person-years, again showing a clear female predominance (14.2 in women vs 3.17 in men).^3^ Despite this variation over time, the dominance of women’s cases remains a consistent feature of SSc epidemiology worldwide.

Interestingly, a recent nationwide study in South Korea involving nearly 10 million individuals found that women had more than a fivefold increased risk of developing SSc compared to men (adjusted hazard ratio = 5.275).^9^ This elevated risk was particularly pronounced in middle-aged adults and individuals without common comorbidities such as hypertension, abdominal obesity, or dyslipidemia.^9^

A north-south gradient has been observed in Europe, with southern countries such as Italy, Spain, and Greece reporting higher incidence and prevalence compared to northern countries like France, the Netherlands, and Norway.^2^ Interestingly, this gradient persists alongside consistent sex disparities: although women are more frequently affected in all regions, the female-to-male ratio may be even more pronounced in southern populations. For example, Italian and Spanish cohorts reported ratios as high as 8.2:1 and 8:1, respectively,^11,15^ compared to lower but still significant ratios of around 4–5:1 in northern Europe.^2,13^ These differences may reflect complex interactions between environmental exposures, hormonal or genetic predispositions, and diagnostic awareness, all of which could influence both disease frequency and sex-related susceptibility.

Race and ethnicity further influence SSc epidemiology. African American women in the United States exhibit higher incidence rates than their white counterparts (2.25 vs 1.28 per 100,000 person-years) and are more likely to develop dcSSc, which is associated with worse outcomes.^2,16^ These disparities likely reflect a combination of genetic susceptibility and structural inequities in healthcare access and delivery.^2,16^

Age and time to diagnosis also influence sex-based patterns in SSc. Women with SSc generally present at a younger age than men. In the abovementioned Latvian study, the average age at disease onset was 46.5 years in women and 50.5 years in men.^13^ A French-Canadian cohort reported similar findings, with average onset at 39.9 years for women and 43.7 years for men.^17^ However, delays in diagnosis may more often affect women, especially when early symptoms like Raynaud’s phenomenon are overlooked or misattributed. In a Spanish study, women were diagnosed later than men despite having earlier symptom onset.^15^ Consistently, in the abovementioned French-Canadian cohort, the overall mean age at diagnosis was 49.4 for women and 48 for men.^17^ Overall, time to diagnosis is typically longer in women, while men tend to receive a diagnosis sooner after the onset of Raynaud’s, likely due to a more rapid and aggressive disease course.^11,15,17–19^

Finally, epidemiological data from juvenile SSc populations confirm the higher prevalence of affected girls compared to boys, with a female-to-male ratio of approximately 4:1, which is less pronounced than in adult-onset SSc.^20^

## Pathogenesis of sex-related differences in SSc

This sex bias in SSc suggests that sex-related pathophysiological mechanisms influence disease susceptibility and expression. The interplay of sex hormones, X chromosome-linked genetic factors, and epigenetic as well as environmental exposures contributes to this disparity.

### Sex hormones and fibrosis regulation

The role of estrogens in immune regulation and fibrotic processes is multifaceted and context-dependent. Rather than exerting uniformly protective or harmful effects, estrogens demonstrate both immunomodulatory and fibrogenic potential, influenced by concentration, receptor subtype, target tissue, and disease state.^21–24^ In the context of SSc, estrogen receptor-α knockout and estrogen inhibition (treatment with tamoxifen) in murine models exacerbated dermal fibrosis, while estradiol treatment attenuated the transforming growth factor beta-driven (TGF-β) collagen synthesis and myofibroblast differentiation.^25^

However, this protective effect is not universal and differs in human studies. A systematic review of the sex hormone levels and sex hormone-targeting therapies in patients with SSc highlighted the possible profibrotic effects of estrogens.^26^ In addition, in clinical settings, elevated estradiol levels in older men with SSc have been associated with increased cardiac involvement, worsening of dermal fibrosis and poorer survival outcomes, emphasizing the complex effects of estrogen signaling in SSc pathogenesis.^27^

Postmenopausal hormonal changes influence the progression and clinical expression of SSc. An Italian retrospective study found that postmenopausal women had a much higher risk of developing isolated pulmonary arterial hypertension (PAH; relative risk = 5.2, *p* = 0.0001), suggesting that estrogen deficiency may contribute to endothelial dysfunction and vascular damage in SSc.^28^ On the other hand, estrogens may worsen skin fibrosis by increasing the production of extracellular matrix proteins. As a result, postmenopausal status has been linked to reduced skin fibrosis in patients with dcSSc, as shown by lower modified Rodnan skin scores. This suggests that estrogen withdrawal may have tissue-specific effects.^29^ Interestingly, data from the Italian SPRING registry highlight that early menopause (before age 45) is linked to a more severe SSc phenotype affecting both the vascular and fibrotic features: patients with early menopause experienced more frequent digital ulcers and interstitial lung disease (ILD).^30^ However, exogenous estrogen exposure may not be protective: menopausal hormone therapy has been linked to an increased risk of developing SSc, particularly when initiated several years before disease onset, raising concerns about the role of prolonged estrogen exposure in disease pathogenesis.^31^

Androgens may also influence the differences in disease expression between sexes in SSc. Interestingly, Perković et al. reported a negative correlation between androstenedione levels and ACA levels in postmenopausal women, suggesting a potential protective role of androgens in lcSSc. In contrast, the same study found a positive correlation between androstenedione and testosterone levels and the presence of anti-Scl-70 antibodies, which are typically associated with dcSSc. These findings point to a potentially opposing effect of androgens depending on the autoantibody profile and disease subtype.^32^

Another hormone that may contribute to the sex-based differences seen in SSc is prolactin. Elevated levels of prolactin, which are more commonly observed in women with SSc, have been linked to increased immune activation and potentially greater disease severity.^33^

Ultimately, these observations point to a central role of sex hormone imbalance—whether due to deficiency, excess, or altered signaling—in modulating disease risk, phenotype, and progression in SSc.

Pregnancy is a period of immune-endocrine changes with implications in SSc pathogenesis. Although no direct evidence links hormonal changes during pregnancy with SSc complications, such shifts may nevertheless contribute to disease expression or outcomes. Patients with SSc face an increased risk of adverse obstetric events, including miscarriage, intrauterine growth restriction and preterm birth. Risk is especially elevated in those with early dcSSc or preexisting organ involvement, which may also predispose to maternal complications such as preeclampsia.^34–37^ While disease activity often remains stable during pregnancy with appropriate management—and some women even report improvement in Raynaud’s phenomenon and digital ulcers (DU)^36^—the observed increase in obstetric risks raises the possibility that hormonal fluctuations could influence disease behavior. In addition, parity has been suggested to influence disease phenotype, with lcSSc and pulmonary fibrosis more common in women with more children.^38^

#### Transgender people

A case series reported the development of SSc in transgender women (assigned male at birth) receiving estrogen-based therapy, showing a range of clinical features, including both limited and diffuse cutaneous involvement, and autoantibodies.^6^ The authors could not rule out a possible link between gender-affirming hormone therapy and the onset of SSc. In contrast, transgender men (assigned female at birth) receiving testosterone are rarely reported in SSc case studies, and there is not enough evidence to draw conclusions about the role of testosterone or gender-affirming hormone therapy in this population. So far, while caution is advised when starting hormone therapy, there are no current recommendations against its use in transgender individuals.^39,40^

### Genetic susceptibility

On top of sex hormones, a growing body of evidence highlights the importance of genetic mechanisms that differ between sexes—that is, those involving the X chromosome, immune gene regulation, and cell signaling pathways.

*X Chromosome inactivation escape* in female immune cells involves certain immune genes located on the X chromosome, leading to biallelic expression and enhanced immune activity.^41^ More specifically, TLR7 and TLR8 genes are overexpressed in plasmacytoid dendritic cells from female SSc patients due to altered nuclear positioning and incomplete X-inactivation, driven in part by reduced expression of X-inactive specific transcript (XIST) and its silencing partner SPEN.^42^ These receptors activate type I interferon production and CXCL4 secretion, both central to SSc pathogenesis.^41–43^ Similarly, IRAK1, a kinase involved in TLR/IL-1 signaling and activation of the transcription factor NF-κB, also escapes X-inactivation and is overexpressed in women, possibly contributing to SSc pathogenesis.^44^ Moreover, CD40LG gene, which encodes CD154 that contributes to T and B cell stimulation, is re-expressed from the inactive X chromosome in CD4+ T cells due to demethylation of its regulatory regions. This results in pathologically elevated CD40L levels in women with SSc, possibly facilitating inflammation.^45^ Recent research has also highlighted the immunological role of the XIST itself. Dou et al. demonstrated that the XIST ribonucleoprotein (RNP) complex—comprising multiple autoantigenic components—can trigger autoantibody formation and autoimmune pathology when ectopically expressed in male mice, promoting T and B cell profiles resembling those of women. Multiple XIST-binding proteins are targeted by autoantibodies in patients with SSc, SLE, and dermatomyositis, suggesting the XIST RNP as a shared and novel immunogenic complex in female-biased autoimmunity.^46^

*Skewed X Inactivation*, a nonrandom preference for silencing one X chromosome, has been reported more frequently in female SSc patients than in controls and is associated with reduced FOXP3 expression and impaired Treg function, weakening immune tolerance and increasing autoimmune susceptibility.^47^

*Genetic variants* also contribute to sex-specific susceptibility in SSc. Recent studies have shown that copy number variation in the complement genes C4A and C4B modifies SSc risk in a sex-dependent manner: higher total C4 dosage is protective, but the effect differs by sex; in fact, C4A offers stronger protection in men, while C4B is more protective in women.^48^ Another locus showing sex-specific effects is Neuropilin-1 (NRP1). A genome-wide interaction analysis identified a variant near NRP1 (rs2812627) with opposite associations by sex—suggesting risk in males but a modest protective trend in women. NRP1 encodes a co-receptor for the vascular endothelial growth factor (VEGF) and regulates endothelial cell function, relevant to vasculopathy observed in SSc. It is also a marker for a subset of regulatory T cells and participates in immune homeostasis and tolerance. Given known sex differences in T cell populations and endothelial biology, the impact of NRP1 on disease risk may reflect both vascular and immunological pathways.^49^

*Epigenetic mechanisms* are central to sex-biased immune dysregulation in SSc. Studies in monozygotic twins have revealed differential methylation in both X-linked and autosomal genes involved in proliferation, apoptosis, oxidative stress, and inflammation, underscoring the role of epigenetic modulation in disease susceptibility independent of genetic variation.^50^ Moreover, the transcription factor VGLL3 has been identified as another sex-biased epigenetic regulator. Expressed at higher levels in female skin, VGLL3 drives the expression of proinflammatory genes implicated in the pathogenesis of SSc. Notably, VGLL3 activity is hormone-independent, further supporting the role of epigenetically regulated factors in shaping autoimmune risk.^51^

### Environmental triggers

Environmental exposures, especially to silica and organic solvents, are known risk factors for SSc, particularly in people with a certain genetic predisposition.^52^ These exposures are more prevalent in certain occupational settings traditionally dominated by men, such as construction or mining, which may partly explain the observed sex-related differences in SSc severity. Similarly, exposure to heavy metals, such as cadmium, lead, and mercury, has been linked to SSc in women, likely reflecting different occupational roles, such as work in electronics, battery production, or manufacturing. These patterns suggest that sex-based differences in workplace exposures may contribute to the distinct clinical presentations of SSc in men and women.^53^

In addition, silicone breast implants, especially ruptured ones, have been linked to the development of SSc associated with anti-RNA polymerase III antibodies in women.^54^

## Clinical and serological differences between sexes

**Figure 1.**
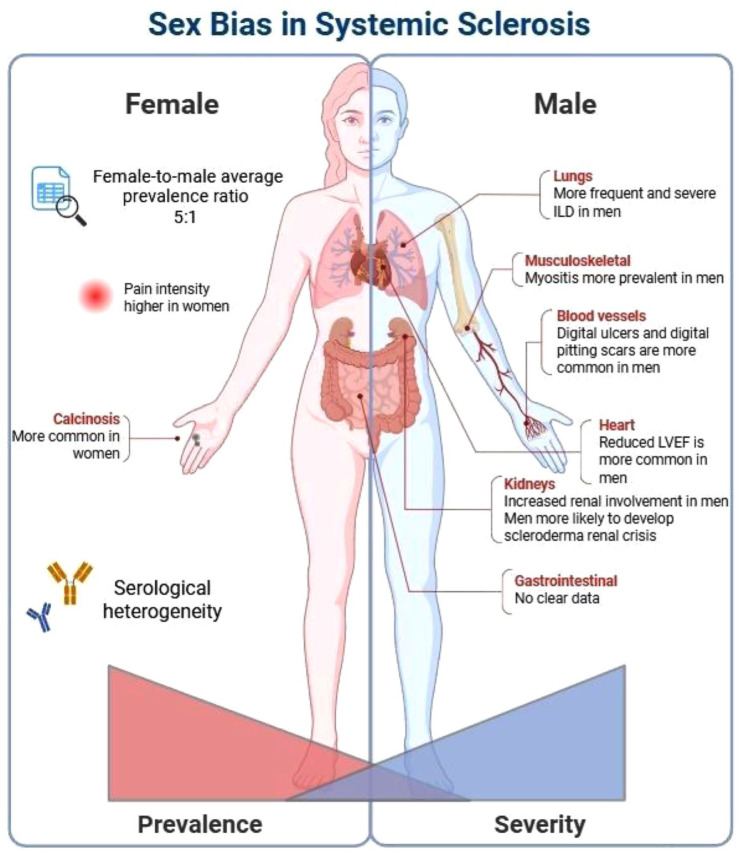
Sex bias in systemic sclerosis. Source: Figure created in Biorender. ILD, interstitial lung disease; LVEF, left ventricular ejection fraction.

Sex differences in the clinical presentation of SSc have been widely studied. While some findings have been inconsistent, there is clear evidence that men are more likely to develop severe disease forms and have worse survival compared to women (Table 1 and Figure 1).

**Table 1.** Sex-based variation in clinical manifestations of systemic sclerosis.

Clinical domain	First author (Ref.)	Registry year	Country and register	Number of patients	Results
Cutaneous subset	De Angelis (11)	2015–2020	SPRING, Italy	2281	F:M dcSSc: 15.2% vs 25.1%, *p* < 0.001
	Peoples (55) Freitas (56) Hussein (57) Elhai (59) Carreira (60)	1985–2011	Pittsburgh Scleroderma Database, USA	2686	F:M dcSSc: 37% vs 48%, *p* < 0.0001
		2008–2020	Reuma.pt./SSc, Portugal	1054	F:M dcSSc: 15.1% vs 34.4%, *p* < 0.01
		1970–2013	Toronto Scleroderma Program, Canada	959	F:M dcSSc: 15.2% vs 25.1%, *p* < 0.001
		2004–2013	EUSTAR	9182	Male sex predictor of dcSSc (OR: 1.68; 95% CI (1.45–1.94), <0.001)
		2004–2008	EUSTAR	1027	F:M very early disease (<1 year) antiScl70: 31% vs 57%, dcSSc: 33.6% vs 60.2%, *p* < 0.0001 F:M early disease (<3 years) antiScl-70: 31% vs 53%, dcSSc: 35% vs 61%, *p* < 0.0001
Peripheral vasculopathy	De Angelis (11) Elhai (59) Panopoulos (68)	2015–2020	SPRING, Italy	2281	DU F:M 18.9% vs 25.7%, *p* = 0.02 DPS F:M 37.8% vs 49.8%, *p* < 0.001
		2004–2013	EUSTAR	9182	Male sex predictor of DU (OR: 1.28 95% CI (1.11–1.47), *p* < 0.001) F:M active pattern at NVC (42.2% vs 49%, *p* < 0.05)
		1995–2011	Grece	231	F:M DU first–third year: 31.5% vs 54.2%, *p* = 0.036
Respiratory involvement	Freire (15) Peoples (55) Hussein (57) Campochiaro (71)	2006–2014	RESCLE, Spain	1506	F:M ILD 42% vs 57%, *p* < 0.001
		1985–2011	Pittsburgh Scleroderma Database, USA	2686	F:M ILD: 39% vs 52%, *p* < 0.0001
		1970–2013	Toronto Scleroderma Program, Canada	959	F:M ILD 33% vs 41%, RR 1.24 95% CI (1.01–1.52)
		2004–2023	EUSTAR	6389	F:M younger age ILD onset 57.2 ± 13.6 vs 55.9 ± 13.1, *p* 0.002 F:M shorter disease duration at ILD onset (months) 121.2 ± 104.4 vs 79.9 ± 81.4, *p* < 0.001
Calcinosis	Nguyen (76)	2006–2009	Association des Sclerodermiques de France (ASF), France	381	F:M 36% vs 21.4%, *p* = 0.036
GI involvement	Elhai (59)	2004–2013	EUSTAR	9182	F:M Stomach symptoms 24.5% vs 19.5, *p* < 0.001 F:M Intestinal symptoms 24.8% vs 18.2%, *p* < 0.001
	Kassamali (75) Roth (83)	1989–2019	Mass General Brigham Hospitals, USA	2101	F:M 47% vs 39%, *p* = 0.006
		2004–2023	EUSTAR	5462	Female sex predictor of progression in GERD-ILD: HR: 1.39 (1.07–1.80), *p* = 0.012

CI, confidence interval; dcSSc, diffuse cutaneous systemic sclerosis; DPS, digital pitting scars; DU, digital ulcers; GERD, gastroesophageal reflux disease; GI, gastrointestinal; HR, hazard ratio; ILD, interstitial lung; NVC, nailfold video-capillaroscopy.

### Cutaneous subset

Large registry-based cohorts consistently show that the dcSSc is more common in male patients.^55–57^ Interestingly, the female-to-male ratio is highest in lcSSc, reaching up to 11.5:1, and lowest in dcSSc, at around 2.6:1, as observed in a French-Canadian cohort by Scussel-Lonzetti et al.^17^

### Serological profiles

The distribution of autoantibodies shows marked sex-related differences.^58^ In an EUSTAR cohort analysis of 9182 patients, anti-Scl70 antibodies were found more frequently in men.^59^ A subsequent EUSTAR study on patients with early disease confirmed that men exhibited higher rates of both anti-Scl70 positivity and dcSSc, especially within the first 3 years.^60^ Anti-RNA polymerase III is also more common in men and is associated with scleroderma renal crisis (SRC) and rapidly progressive skin thickening. Similarly, anti-fibrillarin (anti-U3-RNP) is more frequently found in men of African, Afro-Caribbean, and Native North American descent and is likewise linked to severe disease.^55,58,61–65^ In contrast, ACAs and anti-Ro52 (TRIM21) are more commonly found in women. TRIM21 seropositivity is associated with lcSSc, pulmonary hypertension (PH), ILD, and joint manifestations.^55,58,66^ Anti-NOR90, another rare antibody, shows a female predominance and has been linked to favorable prognosis, lower skin scores, and less gastrointestinal involvement.^67^ Interestingly, ANA-negative SSc patients are significantly more likely to be male and exhibit fewer vasculopathic features, such as PAH, telangiectasias, and DU, but possibly, more frequent lower gastrointestinal involvement.^58^

### Peripheral vasculopathy

Peripheral vasculopathy is a meaningful and potentially severe complication of SSc. A previous study on a homogeneous cohort of 231 patients found that men were more likely to develop DU, as well as SRC, within the first 3 years of disease onset.^68^ These sex-related differences have been corroborated by large EUSTAR cohort analyses, showing male sex as an independent risk factor for DU (adjusted odds ratio 1.28, 95% confidence interval (CI) 1.11–1.47). In addition, men exhibited a more active pattern on nailfold videocapillaroscopy (NVC) compared to women (49% vs 42.2%).^59^ Supporting data from the Italian SPRING registry confirmed the higher prevalence of DU (49.8% vs 37.8%, *p* < 0.001) and digital pitting scars (25.7% vs 18.9%, *p* = 0.02) in men, alongside an increased frequency of SRC (2.1% vs 0.7%, *p* = 0.038).^11^

### Cardiac involvement

A large analysis from the EUSTAR registry on 7073 patients identified male sex as an independent predictor of reduced left ventricular ejection fraction (LVEF < 55%), alongside factors such as older age, longer disease duration, DU, dcSSc, higher disease activity score, pulmonary fibrosis, PAH, renal involvement, and muscle involvement.^69^ These findings were corroborated by a case-control study of 129 patients with reduced LVEF and 256 matched controls, where male sex was independently associated with left ventricular dysfunction (OR 3.48, 95% CI: 1.74–6.98).^68^ Kahan et al. further proposed that the observed link between DU and myocardial impairment may reflect the widespread microvascular dysfunction inherent to SSc in males.^70^ Considering the higher prevalence of microvascular damage in male patients, future studies focusing on the interplay between sex, peripheral vasculopathy, and cardiac involvement may offer important insights into sex-specific pathophysiological mechanisms in SSc.

### Respiratory involvement

Multiple cohort studies consistently report a higher prevalence of ILD in males with SSc. In the larger Pittsburgh Scleroderma Database, including 2686 patients, men were more likely to present ILD (52% vs 39%, *p* < 0.0001) and were also more prone to severe or end-stage lung disease compared to women (53% vs 42%, *p* = 0.0013).^55^ Similarly, the RESCLE Spanish registry showed a higher ILD prevalence in men (57% vs 42%, *p* < 0.001), along with lower forced vital capacity (FVC) values and a greater proportion of cases with FVC <70%.^15^ A recent EUSTAR analysis further supported these findings, showing that ILD tends to develop earlier and with shorter disease duration in men than in women.^71^ While ILD is well studied in SSc, less is known about small airway disease (SAD), which affects airways under 2 mm in diameter. Interestingly, recent findings suggest that SAD is more common in women.^72^ Similarly, PAH has been reported to be more frequent in women but is associated with a higher disease burden and a short interval from SSc-diagnosis in men.^73^

### Gastrointestinal involvement

Findings on gastrointestinal involvement in SSc are mixed, largely due to the wide range of symptoms and differences in how patients are grouped for research. Some studies found no significant differences between men and women,^56,68,74^ while others reported a higher prevalence of GI disease in females (47% vs 39%, *p* = 0.006).^59,75^

### Calcinosis

Calcinosis, the abnormal deposition of calcium in skin and soft tissues, shows signals for being more prevalent in women. Nguyen et al. reported that calcinosis was more common in women (36.0% vs 21.4%, *p* = 0.036).^76^ This supports other findings that link calcinosis more often with the lcSSc, which is more frequently seen in women.^77^

### Musculoskeletal involvement

Overlap with myositis seems to be more prevalent in men than women in the early stages of lcSSc,^60^ as well as in established SSc.^55^ However, a recent review of musculoskeletal involvement in SSc did not highlight any clear sex-based differences for musculoskeletal manifestations.^78^

### Pain and psychosocial burden

SSc patients frequently experience pain due to arthritis, DU and Raynaud’s phenomenon. A study by Lee et al. found that women reported feeling more intense pain than men.^79^ In addition, health-related quality of life (HRQoL), disability, depression, and anxiety were investigated in male and female patients with SSc. Women reported higher levels of anxiety, measured using the Hospital Anxiety and Depression Scale, which is consistent with earlier research.^76^. However, sex did not appear to significantly affect the frequency of depression, perceived disability, or impaired HRQoL, as measured by the 36-Item Short Form Health Survey (SF-36).^76^ These findings suggest that functional and social challenges are equally burdensome for men and women.

## Prognostic implications of sex in SSc

Despite advances in understanding sex differences in the clinical features of SSc, much remains to be explored, particularly regarding sex-based differences in organ-specific disease progression. Most studies have focused on respiratory involvement, with limited research on other domains.

Male sex has been linked to higher systolic pulmonary artery pressure (>35 mmHg) at baseline on echocardiography.^76^ It also contributes to PAH progression after 3 years from the diagnosis^80^ and has been independently associated with new onset of PH (any PH) during the disease course (hazard ratio (HR): 2.66 (1.32–5.36), *p* = 0.006).^59^ Male sex has also been recognized as a risk factor for progressive ILD.^81,82^ As previously noted, men tend to develop ILD earlier in the disease course, at a younger age, and with higher related mortality compared to women.^59^ Supporting these observations, Campochiaro et al. examined ILD progression in the first year of disease. Although some differences were observed, male sex, unlike in the abovementioned studies,^81,82^ did not emerge as an independent predictor of lung function decline.^71^ This discrepancy may reflect differences in the methodological approaches used across studies. Notably, predictors of disease progression appeared to vary by sex. In women, older age was associated with an increased risk of progression, whereas no significant predictors of marked progression were identified in men.^71^

Interestingly, in women, ILD has been more frequently associated with gastroesophageal reflux disease, and sex has been identified as an independent predictor of ILD progression in this context (HR: 1.39, 95% CI: 1.07–1.80, *p* = 0.012).^83^ These findings suggest that disease progression may follow distinct trajectories in men and women across various organ systems.

Regarding cutaneous involvement, Carreira et al. found that in early stages of SSc, 12 and 36 months after the onset of the first non-Raynaud’s phenomenon sign or symptom, men had higher inflammatory markers and more frequent muscle and lung involvement compared to women. This is particularly evident in male lcSSc patients, who exhibit a more severe clinical presentation in the early phase of the disease, characterized by increased organ involvement, lower ACA levels, and higher inflammatory indices. However, men and women with early dcSSc had similarly severe disease.^60^

In terms of mortality, several studies have shown higher death rates in men.^84–86^ In a large Spanish cohort, the 10-year survival rate was 75.3% in men compared to 92.9% in women.^15^

Similarly, a French cohort of over 2000 SSc-related deaths reported a female-to-male ratio of 3.8.^86^ While male sex has been identified as a predictor of all-cause mortality, it was not associated with SSc-specific mortality in an EUSTAR cohort.^59^ However, male sex has been linked to worse outcomes in patients with SSc-ILD, SSc-PAH, and SSc-PH (or at risk for PH).^71,87,88^

## Sex-specific treatment effects

Current international guidelines do not provide sex-specific treatment recommendations for SSc.^89^ However, sex influences clinical presentation and outcomes, as mentioned above. Regarding prescription choices, treatment efficacy, and side effects, existing data are limited and primarily derive from post hoc analyses.

### Prescription choices

In real-world data from Canada and Germany, no significant differences were observed between the sexes in the prescription of immunosuppressive or vasoactive therapies.^90,91^ One study reported that methotrexate is less frequently used in female SSc patients, likely due to its teratogenic properties.^57^ However, more robust data on this topic is needed.

### Treatment efficacy

Post hoc analyses of the SLS I and II trials provided further insights into sex-related differences in treatment efficacy among SSc-ILD patients. First, female patients demonstrated better treatment responses to mycophenolate mofetil (MMF) and oral cyclophosphamide (CYC), as measured by FVC changes. In the CYC treatment group, adjusted for baseline ILD severity, male participants had a decline of FVC within the first year, whereas female participants had an improvement of FVC (estimated effect = −0.72, *p* = 0.0006). Concerning MMF treatment, improvement of FVC was observed in male and female participants, but the rate of improvement tended toward superiority in women (estimated effect −0.34, *p* = 0.0051). In the pooled data analysis of CYC and MMF treatment, FVC decline of 10% or greater within 1 year was more frequent in men (10%, *n* = 5/48 participants) versus women (5%, *n* = 7/134 participants).^92^

Similar data were confirmed in a post hoc analysis from the FocuSSed trial, combined with data from a large SSc cohort (SMART), found that male sex was associated with a more favorable response to tocilizumab, as indicated by a reduced rate of FVC decline ⩾10%.^82^

Interestingly, a posthoc analysis from the SENSCIS trial showed a numerically greater benefit of nintedanib in men compared to women (FVC decline −58.6 mL/year vs −34.6 mL/year), without confirmation of significance in interactive *p*-value (*p* = 0.59).^93^. Yet, FVC decline in the placebo group was more pronounced in male compared to female patients (−126.8 mL/year vs −82.0 mL/year), which might support the aforementioned difference. These differences in treatment responses might be explained by a more progressive course of ILD in males,^82,91^ making it easier to detect a treatment effect. In addition, differences in pathogenesis according to sex may also play a role.

Indeed, the analysis of bronchoalveolar lavage fluid revealed distinct immune profiles according to sex: men exhibited significantly higher concentrations of profibrotic cytokines (MMP13 and TIMP1), while women showed increased levels of proinflammatory cytokines (IL-7 and IL-12). These findings support the hypothesis of sex-specific immune pathways and disease mechanisms.

### Side effects

Pharmacokinetics, which differ between male and female individuals, play a crucial role in determining side effects. Due to differences in weight, height, and volume of distribution, women are often exposed to higher blood drug concentrations, potentially leading to altered drug levels compared to men.^94^

There is limited data on this topic. One study investigated side effect profiles of SSc treatments. As reported by Hoffmann-Vold et al., nintedanib showed comparable side effects between male and female patients.^95^ However, nausea and elevated liver enzymes appeared more frequently in women.

With the growing number of available treatment options for SSc, medical adherence has become an increasingly important area of investigation. While medical adherence was demonstrated to be impacted by treatment satisfaction, polypharmacy and patients’ disease perception, studies did not indicate significant sex-based differences in adherence among SSc patients.^96^ Similarly, in other chronic autoimmune diseases such as rheumatoid arthritis and systemic lupus erythematosus, no consistent sex-related differences in adherence have been observed.^97,98^

## Gender

While sex differences in SSc have been partly studied, the role of gender in this context has not yet been a focus of research. The terms “sex” and “gender” are often misunderstood, and it is important to distinguish between them: sex refers to biological characteristics such as hormones, chromosomes, and reproductive anatomy, whereas gender is a multidimensional construct encompassing social, cultural, and psychological aspects, including social roles and relationships. Given the significant differences in prevalence and outcomes between men and women in SSc, shifting the focus from sex to gender may provide novel insights. Despite this potential, gender remains an unexplored factor in SSc research.

## Conclusion

Understanding how sex influences the course of SSc has revealed important biological and clinical differences, such as the higher prevalence in women and more severe organ involvement in men. Recent advances have started clarifying the mechanisms behind these patterns. However, despite this growing knowledge, sex-specific insights have yet to be incorporated into clinical guidelines or treatment strategies. Future studies should integrate both biological sex and gender dimensions to capture the full spectrum of disease variability. Moving forward, incorporating sex- and gender-specific considerations into clinical trials and patient care may lead to more equitable and effective strategies for managing SSc.

## Declarations

**Ethics approval and consent to participate:** Not applicable.

**Consent for publication:** Not applicable.

**Availability of data and materials:** Not applicable.
